# Physiological links of circadian clock and biological clock of aging

**DOI:** 10.1007/s13238-016-0366-2

**Published:** 2017-01-20

**Authors:** Fang Liu, Hung-Chun Chang

**Affiliations:** 1 Institute of Neuroscience, State Key Laboratory of Neuroscience, Key Laboratory of Primate Neurobiology, CAS Center for Excellence in Brain Science and Intelligence Technology, Shanghai Institutes for Biological Sciences, Chinese Academy of Sciences, Shanghai, 200031 China; 2 University of Chinese Academy of Sciences, Shanghai, 200031 China

**Keywords:** circadian rhythms, SCN, longevity

## Abstract

Circadian rhythms orchestrate biochemical and physiological processes in living organisms to respond the day/night cycle. In mammals, nearly all cells hold self-sustained circadian clocks meanwhile couple the intrinsic rhythms to systemic changes in a hierarchical manner. The suprachiasmatic nucleus (SCN) of the hypothalamus functions as the master pacemaker to initiate daily synchronization according to the photoperiod, in turn determines the phase of peripheral cellular clocks through a variety of signaling relays, including endocrine rhythms and metabolic cycles. With aging, circadian desynchrony occurs at the expense of peripheral metabolic pathologies and central neurodegenerative disorders with sleep symptoms, and genetic ablation of circadian genes in model organisms resembled the aging-related features. Notably, a number of studies have linked longevity nutrient sensing pathways in modulating circadian clocks. Therapeutic strategies that bridge the nutrient sensing pathways and circadian clock might be rational designs to defy aging.

## INTRODUCTION

With seminal successes in biomedical researches, the improved medical conditions markedly lengthened human lifespan however also led to emerging threats as known the age-associated complexities (Kaeberlein et al., 2015). The wide range of age-associated diseases, including neurodegenerative diseases, cardiovascular disorder, type-2 diabetes, and higher cancer incidences, are driven by the causes of time, genetic and environmental situations that remain difficult to dissect for major effector(s) in individuals. Over decades, researches on aging have revealed the retardation of physiological decline and lifespan extension are conceivable by genetic perturbations in model organisms, the results now offered potential therapeutic strategies to prolong both healthspan and lifespan (Lopez-Otin et al., 2016). Dietary restriction (DR), a chronic reduction of dietary intake regime was proven as a major link of connecting these genetic longevity studies. DR increases lifespan in many model organisms, including budding yeast *Saccharomyces cerevisiae*, nematode *Caenorhabditis elegans*, and fruitfly *Drosophila melanogaster*. These relatively simplified models rendered further analyses of longevity genes and pathways that are activated upon low-energy challenges thus mimicked DR effects (Fontana and Partridge, 2015; Guarente, 2013). Importantly, salutary effects of DR is evolutionarily conserved as also observed in primates (Colman et al., 2009; Colman et al., 2014). The significance of DR emphases the idea that energy homeostasis is centered in longevity, while aging is largely caused by aberrant energy condition and metabolic inflexibility (Riera and Dillin, 2015). Thus interactions among calorie intake, meal frequency and timing, as organized by the daily circadian rhythm program, are likely key to maintain the cellular and organ fitness.

Circadian rhythms govern a wide range of physiological and behavioral systems, such as energy metabolism, sleep-wake cycles, body temperature and locomotor activity (Panda et al., 2002; Reppert and Weaver, 2002). Declined circadian rhythmicity in endocrine rhythm, phase alignment and sleep are commonly seen with aging (Mattis and Sehgal, 2016). Consistently, experimental disruptions of circadian rhythms seriously impede functional physiology, lifespan and endorse cancer incident (Filipski et al., 2003; Froy, 2013; Fu et al., 2002; Kondratova and Kondratov, 2012; Penev et al., 1998). Even a milder circadian challenge, chronic jet-lag, imposes on aged wild-type mice can markedly increase mortality (Davidson et al., 2006). On the other hand, implant functional circadian clock with fetal suprachiasmatic nucleus in aged rodents allowed higher amplitude rhythm behavior and longer surviorship (Hurd and Ralph, 1998; Li and Satinoff, 1998). The evidence pictured the pivotal contributions of robust circadian rhythms in upholding the healthy physiology and likely the extension of lifespan.

This review includes an overview of the molecular mechanism of circadian control, and molecular deficiencies implicated in age-related malfunctions. It discusses the central circadian clock system and the pathologies with aging, including the impacts to neurodegenerative diseases and sleep. Finally, advises the links of circadian components to energy-sensing pathways that modulate mammalian lifespan, furthermore their potential as therapeutic targets to treat age-associated loss in physiological homeostasis.

## MOLECULAR OSCILLATORS IN THE CIRCADIAN CLOCK

**Figure 1:**
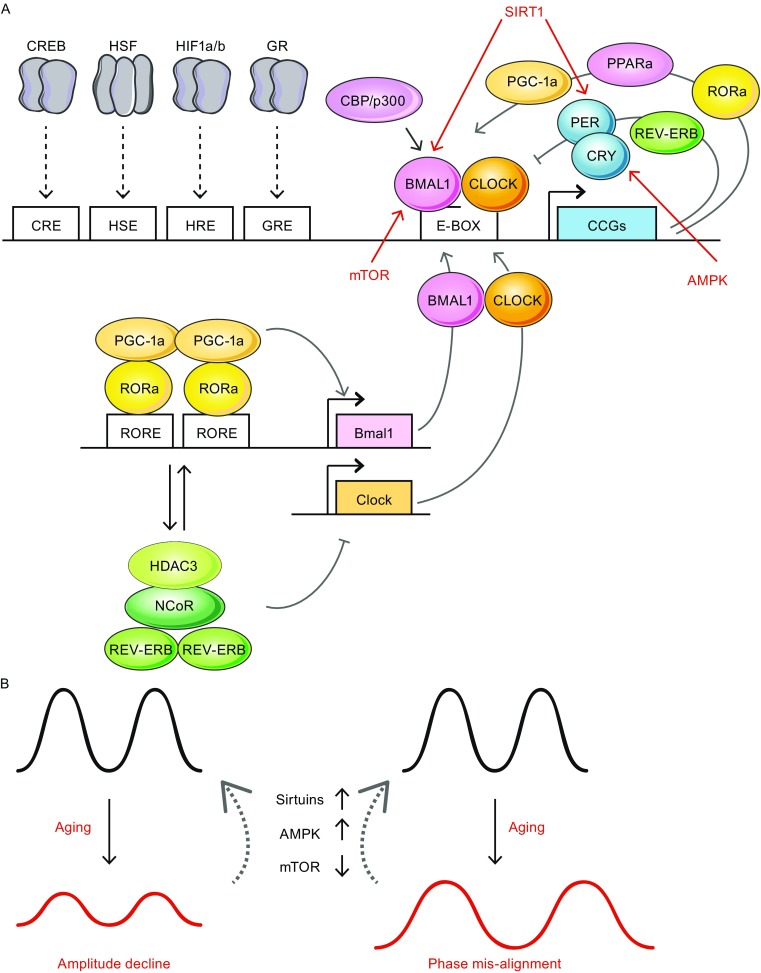
**Molecular oscillators in circadian control.** (A) Transcription factor complex CLOCK:BMAL1 binds to E-box containing motifs, allows the transcriptional activation of clock-controlled genes (CCGs) such as *Pers*, *Crys*, *Ror* and *Rev-Erb*. The activation is facilitated by recruiting coactivators such as CBP/p300. CCG transcriptions are as well regulated by transcription factors relaying the external cues. Examples include cAMP responsive element binding protein (CREB), heat shock factor 1 (HSF1), hypoxia-inducible factor 1α (HIF1α) and glucocorticoid receptor (GR) that bind to their respective regulatory elements (Bollinger and Schibler, 2014; Wu et al., 2016). Two interconnected feedback loops involved in the circadian transcriptional regulation. In the primary feedback loop, PER and CRY assemble into repressor complexes next attenuate the activity of CLOCK:BMAL1. In the second feedback loop, ROR (also a CCG protein) can complex with coactivator PGC-1α and bind to RORE element for *Bmal1* (and likely *Clock*) activation(s). REV-ERB works as a repressor in *Bmal1* transcription by concentration-dependent competition at the same RORE sequence. The repression involved the recruitment of NCoR/HDAC3 corepressor complexes. (B) Energy sensors such as Sirtuins, AMPK and mTOR participate in circadian modulations via post-translational modification of circadian components, as depicted in (A). Interventions target the pathways are of potential to treat age-associated circadian amplitude decline and phase mis-alignment

Circadian oscillations are generated via transcriptional–translational feedback loops in a cell autonomous manner in mammals (Bass and Takahashi, 2010; Dibner et al., 2010). The core transcription factors CLOCK and BMAL1 heterodimerize and bind to E-box motif-containing clock-controlled genes (CCGs) in a time-dependent manner. There are at least two interconnected feedback loops involved in the transcriptional regulation (Fig. 1). In the primary feedback loop, CLOCK:BMAL1 initiates the transcription of *Period* (*Per*) and *Cryptochrome* (*Cry*) through the binding of E-box elements. The transcriptional control is also facilitated by recruiting various coactivators including CBP/p300 (Hosoda et al., 2009; Li et al., 2010), TRAP150 (Lande-Diner et al., 2013) and SRC-2 (Stashi et al., 2014). When CRYs and PERs proteins accumulate to critical levels, they assemble into hetero-complexes and function as corepressors via direct binding to CLOCK:BMAL1 thus repress their own expression. The repression is facilitated by posttranslational modifications, for instance phosphorylation of PERs for nuclear translocation hence binding to CLOCK:BMAL1 (Lee et al., 2001). The repression is later relieved by the degradation of CRYs and PERs over time, allows another circadian cycle of CRYs and PERs expressions taking place. In the secondary loop, the nuclear orphan receptors REV-ERBα, REV-ERBβ, RORα, RORβ and RORγ are involved in controlling the temporal expression of BMAL1 and CLOCK. Of note, these nuclear orphan receptors are also CCGs under CLOCK:BMAL1 regulation. By recognizing RORE elements within the promoters of *Bmal1* and *Clock* genes, ROR collaborates with PGC-1α to transcriptionally activate *Bmal1* and *Clock*. REV-ERB competes for the RORE binding at circadian times with concentration advantage over ROR, and executes as BMAL1 and CLOCK repressor (Cho et al., 2012; Preitner et al., 2002; Sato et al., 2004). The repressing activity requires the recruitment of a NCoR1-HDAC3 corepressor complex (Everett and Lazar, 2014). Recent findings of hypoxia-inducible factor 1α (HIF1α) in additional regulatory route indicated oxygen level is an auxiliary cue in clock. HIF1α itself is a CCG and at hypoxia, activates per expression via binding to the hypoxia-responsive element (HRE) in a complex with ARNT. The results are of clinical interests to cardioprotection and phase conditions such as jetlag (Adamovich et al., 2017; Peek et al., 2017; Wu et al., 2017). Other transcriptional regulations in particular cell types, for instance ZBTB20 in rhythmic expression of prokineticin receptor-2 (*Prokr2*) in the suprachiasmatic nucleus neurons, is critical for the bimodal activity behavior in mice (Qu et al., 2016). It will be important to decipher known, or identify new regulatory mechanisms in cell types that are responsible for unique circadian behaviors.

Notably, both the primary and secondary feedback loops are modulated by post-translational modifications in versatile ways, e.g, protein ubiquitination, phosphorylation/dephosphorylation, acetylation/deacetylation, poly ADP-ribosylation and O-GlcNAcylation (Reddy and Rey, 2014). These modifications indicate evident basis linking circadian and metabolic cycles at timely manner. Identify new post-translation modifications and classify the modifications in the central and peripheral tissues will be of great value to understand circadian physiology.

## CLOCK GENES AND AGE-RELATED DISORDERS

Aging is a major risk factor for many human pathologies, including cancer, diabetes, cardiovascular disorders and neurodegenerative diseases (Lopez-Otin et al., 2013). Genetic models of circadian disruption pheno-copied aging and metabolic disorders frequently. A prominent case is the loss of BMAL1. Mice deficient for *Bmal1* are suffered from a series of conditions related to aging. e.g., sarcopenia (with both reduction in muscle fiber size and quantity), cataracts, cornea inflammation, osteoporosis, premature hair loss, and failed to form adequate visceral and subcutaneous adipose storage (Kondratov et al., 2006). The strain is severely short-lived with average lifespan of 37.0 ± 12.1 weeks, compared to longer than 110 weeks of lifespan in same background wild type animals (Nadon, 2006). The findings coordinate well with the roles of BMAL1 in homeostatic maintenance of the glucose level (Rudic et al., 2004), and in adipogenesis regulation (Shimba et al., 2005). Consistently, it has been noticed that *Bmal1* mRNA amplitude declined with altered peak phase in natural aging in rodents (Kolker et al., 2003).

As a reciprocal component of BMAL1, CLOCK deficiency also results in shorter average lifespan to approximately 15% reduction compared to wild type, and premature pathologies including cataracts and dermatitis (Dubrovsky et al., 2010). CLOCK appears to be crucial in glucose homeostasis as well, as both whole body and conditional disruptions of CLOCK caused hypoinsulinaemia hence diabetes mellitus in rodents. Same study demonstrated BMAL1 is also participated in sustaining the pancreatic clock (Marcheva et al., 2010). Of note, *Clock∆19* strain, the CLOCK truncated line that was originally identified for its significant period change from a random mutagenesis screen, is with milder aging phenotypes such as diurnal activity/feeding rhythms and obesity in normal housing conditions compared to the knockout strain (Turek et al., 2005). Additional challenges such as post ionizing irradiation triggered an accelerated aging program in the strain (Antoch et al., 2008). The results suggested that the particular CLOCK truncation might be partially functional in protecting from premature aging, at a condition that the intrinsic period is far from optimal. Loss of PER2, a core circadian component, is linked to cancer predisposing. The animals are sensitive to γ irradiation later developed salivary gland hyperplasia, teratoma and malignant lymphomas (Fu et al., 2002). Further, genetic ablation of both *Per1* and *Per2* caused an arrhythmic phenotype together with premature aging conditions, e.g., early decline in fertility, kyphosis and predisposed tumor incidences (Lee, 2005). The DNA damage response and p53-mediated apoptosis are defective in these animals. The studies demonstrated that circadian clock components are also important regulators in cell cycle and proliferation likely specific in adulthood, as the double knockouts seem developmentally normal at birth. Another component CRY1 is shown to modulate hepatic gluconeogenesis by regulating the cAMP signaling. Rhythmic expression of CRY1 directly adjusts intracellular cAMP concentrations and the phosphorylation level of cAMP response element-binding protein (CREB) by protein kinase A (Zhang et al., 2010). Lipid metabolism is linked to circadian clock in the cases of REV-ERB and ROR families. They are important for regulating lipogenesis, lipid storage and adipocyte differentiation in a rhythmic manner (Bray and Young, 2007; Chawla and Lazar, 1993; Torra et al., 2000). REV-ERBs act as decent targets in treating obesity. The agonists work against fat mass accumulation in high fat fed mice, consequently improve dyslipidemia and hyperglycemia (Cho et al., 2012; Solt et al., 2012).

The role of circadian genes in Cancer Biology remains to be a complicated conundrum. As contrast to the tumor suppressing effect of PER2, deletion of *Cry1/2* in p53 null mice protected the early onset of cancer incidence, and sensitized the p53 deficient cells to apoptosis upon genotoxic stress (Ozturk et al., 2009). A recent finding of targeting BMAL1 and CLOCK for acute myeloid leukemia (AML) therapy indicates further the pro-cancerous option of clock components (Puram et al., 2016). Many core circadian proteins are involved in the cell cycle and the DNA damage response (Sahar and Sassone-Corsi, 2009), thus may facilitate the proliferation of transformed malignant cancer cells while normal post-mitotic cells should be exempted from the risk. Careful analyses of cancer types and the associations to circadian gene alterations are essential to address the paradox.

## THE CENTRAL CIRCADIAN CLOCK SYSTEM

To organize physiology and behavior for proper functioning according to the 24-hour environmental light/dark cycle, mammals rely on a central pacemaker known as the suprachiasmatic nucleus (SCN) for systemic synchronization. SCN resides at the anterior hypothalamus and directly contacts optic chiasm for sensing the external photic input. It is composed by paired nuclei lateral to either side of the third ventricle (Colwell, 2011). Though with limited neurons (~20,000 in mouse), SCN contains considerable neuron heterogeneity. There includes calretinin, neurotensin (NT), gastrin releasing peptide (GRP), angiotensin II, prokineticin 2 (PK2), neuromedin S (NMS), vasoactive intestinal peptide (VIP) and arginine vasopressin (AVP) expressing neurons (Welsh et al., 2010). Among them VIP and AVP neurons are key neuron types that mark the ventral core and dorsal shell subdivisions of SCN, respectively (Golombek and Rosenstein, 2010). Most SCN neurons are GABAergic (Moore and Speh, 1993; Morin et al., 2006).

**Figure 2:**
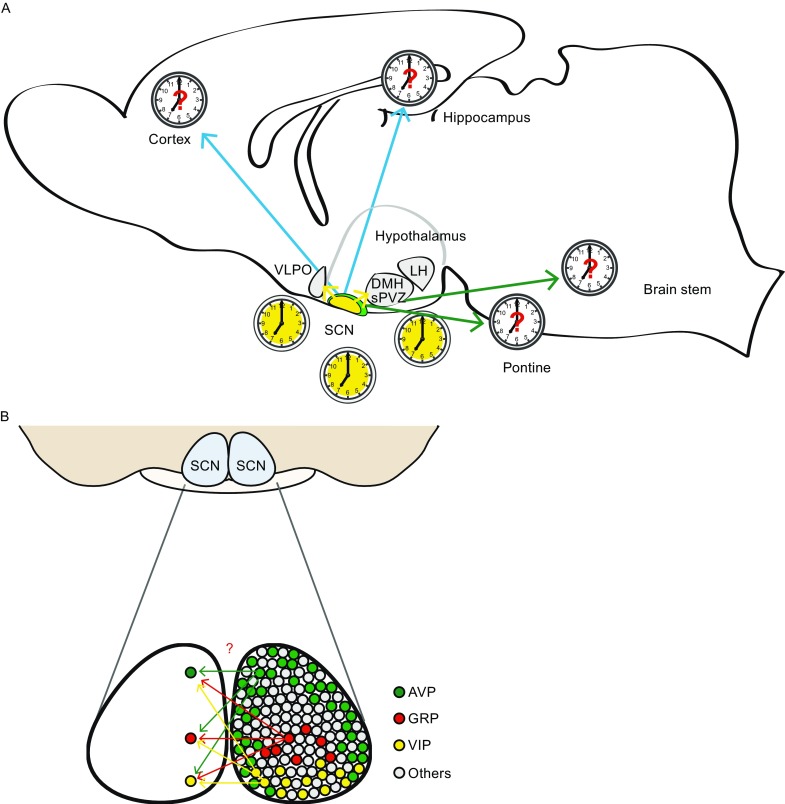
**Schematic functional map of circadian control.** Studies have demonstrated the SCN efferent primarily travel to other hypothalamic nuclei incuding dorsomedial hypothalamus (DMH), subparaventricular zone (sPVZ) and more. A map of direct SCN neuron projections to sleep-awake or cognitive centers in the brain (A) and the intra-SCN (bilateral) connectomes (B), with the details in connection density and neuron types, remains elusive at the moment

Neurons in the core are considered to incorporate external inputs, such as photic light cue from the retinohypothalamic tract (RHT), and likely also the projections from the raphe nuclei (Morin and Allen, 2006). The environmental information is then coupled and communicated to the rest parts of the SCN (Fig. 2). Among the core neurons, VIP neuron is essential in the SCN oscillation coupling; likewise GRP, NT neurons and neurotransmitter GABA contribute significantly to the process as well (Aida et al., 2002; Choi et al., 2008; De Jeu and Pennartz, 2002; Meyer-Spasche et al., 2002; Shinohara et al., 2000). The sensory core neurons display lesser clock gene expression amplitudes, perhaps is suitable for faster resetting when respond to environmental changes, as has been predicted in mathematical modeling work (Pulivarthy et al., 2007). This is by contrast to AVP, PK2, and even GABAergic neurons in the dorsal shell SCN that circadian genes including *Per1* and *Per2* are robustly oscillated in the subdivision (Hamada et al., 2004; Nakamura et al., 2005; Yan and Okamura, 2002).

It has been observed that most of the projections from the core terminate within the shell, stresses the point that the interplay between the two regions is most important among all circadian outputs from the SCN (Antle and Silver, 2005). Several studies have demonstrated the SCN efferent from both core and shell travel chiefly to other hypothalamic nuclei, e.g., dorsomedial hypothalamus (DMH), paraventricular hypothalamic nucleus (PVN), arcuate hypothalamic nucleus (ARC), subparaventricular zone (sPVZ), and more (Abrahamson and Moore, 2001; Kalsbeek et al., 2006; Yan et al., 2007). The projections cover the nervous and endocrine systems for temporal control of the daily oscillation in the body (Dibner et al., 2010). Notably, the projections to central sleep system, for example the ventrolateral preoptic area (VLPO), locus coeruleus (LC) and lateral hypothalamus (LH), are suggested as indirect or sparse (Abrahamson et al., 2001; Aston-Jones et al., 2001; Chou et al., 2002; Novak and Nunez, 2000). This postulates either the sparse connections are sufficient for temporal cues to structure the sleep program, or there exists a central hub, such as DMH, for the communications in between (Chou et al., 2003; Mattis and Sehgal, 2016). A thorough SCN map with neuron type accuracy via connectome works will be of great help to elucidate the functional circadian circuitry (Fig. 2).

## AGE-ASSOCIATED DECLINE IN CENTRAL CIRCADIAN SYSTEM

Important features of functional circadian rhythms include, e.g., sustaining at a sufficient oscillation amplitude through out the daily cycle; composing a phase that is properly aligned with the light/dark condition and can be entrained by light; and maintaining in a near 24 h period to reflect the Earth day (Bass and Takahashi, 2010; Welsh et al., 2010). Aging hampers amplitude both in circadian gene expressions (Hofman and Swaab, 2006; Yamazaki et al., 2002), and several physical indexes including melatonin level, sleep-wake disruptions, lowered locomotor activity (Duffy and Czeisler, 2002; Valentinuzzi et al., 1997; Weinert, 2000; Yoon et al., 2003). Further, phase shifts and re-entrainment difficulty are also common drawbacks with aging (Gibson et al., 2009; Scarbrough et al., 1997; Valentinuzzi et al., 1997). While many factors account for these physical changes, the central clock SCN is likely to stand as a key element responsible for this age-related decline.

The central clock SCN decay, considering the direct or indirect contacts to variable brain regions, would reveal degeneration in at least two aspects: the SCN projections, and secreted signals from the SCN. While projection details of young versus old await careful investigations, SCN secreting outputs have been studied. For example, aging affects SCN prominently in the AVP neuron population. In human, diurnal oscillation of the neuropeptide in young is evident, but in elderly people (over 50 years of age) the change becomes subtle. Further, the peaking time in the early morning in young is reversed to low-amplitude night peaking in the elderly (Hofman and Swaab, 1994). Interestingly, the annual cycle of AVP expression is also lost with aging. Young subjects are normally with lowest AVP-immunoreactive values during the summer and highest in autumn (Hofman and Swaab, 1995). These results suggest that the activities of human SCN, both for the diurnal and the seasonal rhythms, become disturbed later in life. VIP neuron is another, perhaps more sensitive, example to reflect human SCN aging. In young male subjects (10–40 years), the number of VIP neurons in the SCN is highest. However in the age of 40–65 years old, the VIP neuron number dramatically decreased by about 60% further does not show significant decline in later ages (Hofman et al., 1996; Zhou et al., 1995).

Studies in rodents offered further evidences for SCN activity change. First, the AVP neuron is significantly reduced in aged rats while the total SCN neuron number stayed similar (Roozendaal et al., 1987), a change reminiscent to the human case. SCN neurons become desynchronized and are with decreased phase coherence with aging (Farajnia et al., 2012). *In vivo* multiunit neural activity (MUA) recordings in the SCN in young (3–5 months) versus aged (13–18 months) mice revealed that the day and night amplitude differences in the older are significantly reduced. Similar decline in neural activity rhythms was also observed in the subparaventricular zone, one of the major SCN output regions (Nakamura et al., 2011). Whether molecular clock components are good indicators for SCN aging remain unclear. Nakamura et al. applied PER2 as the molecular marker and only revealed subtle change in the two groups, suggested the electrical activity rhythm is the more sensitive circadian output measurement compared to molecular components of the clock. Of note, other experiments showed significant age-related decline of BMAL1 and CLOCK in the SCN as well in few other brain areas, when compared 4 versus 16 month-old mice (Wyse and Coogan, 2010). Further characteristic analyses on SCN aging are crucial to reason the basis of circadian dampens.

## CIRCADIAN DISRUPTION AND NEURODEGENERATIVE DISEASES

Circadian disorders with sleep symptoms are commonly seen in patients with neurodegenerative diseases (Kondratova and Kondratov, 2012). For instance, Parkinson’s disease (PD) patients are disrupted for the cortisol and melatonin rhythms (Breen et al., 2014; Videnovic et al., 2014), and displayed *Bmal1* reduce in blood samples from PD patients (Cai et al., 2010), these all point toward the deteriorated situations in circadian control. A PD transgenic model via *Thy*-1 promoter mediated α-synuclein over-expression exhibited several circadian phenotypes in aging: a clear reduced wheel-running activity with altered period, altered temporal distribution of sleep, and decayed spontaneous neural activity in the SCN, suggested circadian rhythm is severely disrupted in the PD model (Kudo et al., 2011).

Another PD model also links phenotypes to circadian impairments. The strategy is to selectively inactivate mitochondrial transcription factor A (Tfam) in dopamine neurons thus mimics PD progression particularly for dopamine neuron degeneration. The conditional knockouts have reduced physical activity as early as by 5 months of age, and then dampened for both circadian amplitude and stability. The animals also showed abolished rhythmic locomotion in constant dark or constant light conditions (Fifel and Cooper, 2014). Besides circadian phenotypes, sleep disturbance is another hallmark during PD progression. Several sleep disorders are discovered in PD patients, including insomnia, restless leg syndrome and REM behavior disorder (RBD), a symptom that permits motor activity during REM sleep (Barone et al., 2009; Iranzo, 2013). RBD is potentially useful for the prediction of PD onset, as PD pathology occurs in the brainstem earlier than the substantia nigra (Braak et al., 2004). Loss of hypocretin neurons in the lateral hypothalamus could also explain the malfunction of sleep/arousal program in PD patients (Fronczek et al., 2007).

Alzheimer’s disease (AD) patients are long recognized for SCN neuronal loss, of that VIP neuron is a prominent case (Swaab et al., 1985; Zhou et al., 1995). A recent analysis of actogram associated to post-mortem brain tissue demonstrated that besides locomotor activity phenotype, AD patients are also diagnosed with false rhythmic control of core-body temperature and rest-activity (Satlin et al., 1995; van Someren et al., 1996). Sleep–wake cycle dysfunction and increased daytime sleepiness are regard as risk factors for AD related dementia (Lee et al., 2007). Consistently, the transgenic 3xTg-AD mouse strain that exhibits both Aβ and tau pathology (as in human AD) were scored for similar circadian phenotypes. The results indicated that prior to AD pathology, activity during daytime and period change were observed in the transgenic, interesting more in the males. The number of VIP neuron is decreased in the SCN, suggested again that circadian dysfunction is predictive in early AD onset (Sterniczuk et al., 2010a; Sterniczuk et al., 2010b).

Patients with Huntington’s disease (HD) have sleep symptoms including advanced sleep phase, insomnia and reduced REM sleep (Arnulf et al., 2008; Goodman and Barker, 2010). Neuropathological analyses demonstrated that HD patients are depleted for many hypothalamic neuropeptides, i.e., AVP, oxytocin and hypocretin, hence disturbed regular sleep/awake daily cycle (Aziz et al., 2008; Gabery et al., 2010). The HD model, R6/2 transgenic strain displayed reduced expression of *Per2* and blunted oscillation of *Bmal1* in the SCN, as well reduced in motor cortex and striatum. The increased daytime activity is likely associated with reduced prokineticin 2 expression that is critical for suppressing daytime activity in nocturnal animals (Morton et al., 2005). VIP and VPAC2 receptor are down regulated in R6/2 animals in the SCN (Fahrenkrug et al., 2007).

Together, the findings indicate circadian parameters can serve as the basis for prognostic purposes. Sustaining efficient circadian activities are likely key to prevent age-related disorders, including neurodegenerative diseases.

## LONGEVITY MEDIATORS IN CIRCADIAN REGULATION

Vast amount of evidences have pointed out the importance of circadian rhythm in functional physiology, however data that are suggestive to longevity remain unclear. Our current understandings mostly based on loss-of-function studies (Eckel-Mahan et al., 2013), while genetic manipulation of circadian gene has yet been reported with lifespan extension outcome. Interestingly, many longevity mediators and pathways exert the beneficial effects via cooperating with multiple circadian components. One evident example is *Sirt*1, a longevity gene that mediates calorie restriction (CR) benefits (Guarente, 2013), is involved in circadian regulation (Chang and Guarente, 2014; Jung-Hynes et al., 2010). SIRT1 is a NAD^+^-dependent deacetylase that is involved in regulating circadian gene transcriptions via deacetylating histone H3 K9/K14 at the promoters regions (Nakahata et al., 2008). It had been suggested that CLOCK works as a histone acetyltransferase (HAT) in BMAL1 acetylation to facilitate rhythmic circadian gene transcriptions (Hirayama et al., 2007). SIRT1 appears to counterbalance the BMAL1 acetylation status in both fibroblast culture and the liver (Nakahata et al., 2008). Alternatively, SIRT1 deacetylates PER2 thus regulates PER2 stability further adjusts the circadian feedback inhibition (Asher et al., 2008). Notably, the synthesis of SIRT1 cofactor NAD^+^ also follows a circadian expression pattern. Nicotinamide phosphoribosyltransferase (NAMPT), the rate-limiting enzyme for NAD^+^ salvage pathway, is a rhythmically expressed protein that under E-box transcriptional control (Nakahata et al., 2009; Ramsey et al., 2009). Together with facts that SIRT1 oscillates in a circadian manner, and the level of NAD^+^ declines with aging (Gomes et al., 2013), SIRT1 in the interconnected loops revealed a strong correlation between energy and circadian rhythms.

SIRT1 has been demonstrated for numerous vital roles in upholding neuronal health, including neuronal development, memory formation and neurodegenerative disease preventions (Herskovits and Guarente, 2014). Owing to the critical function of hypothalamus in metabolic regulations, many SIRT1 related studies have been carried out in different hypothalamic nuclei. For instance, SIRT1 activities are important for POMC neuron in the ARC, and for SF1 neuron in the VMH to maintain systemic glucose homeostasis hence prevent obesity (Ramadori et al., 2011; Ramadori et al., 2010). A transgenic strain overexpressing SIRT1 in DMH and LH showed improved sleep quality with extended lifespan (Satoh et al., 2013). In the SCN, SIRT1 prevents age-associated circadian phenotypes via supporting molecular oscillation of clock genes (Chang and Guarente, 2013). Of note, SIRT6 (a SIRT1 homolog) also participates in circadian regulation of fatty acid and cholesterol metabolism (Masri et al., 2014). Whether new post-translational modifications take place in circadian modulation, perhaps link to the versatile activities found in other sirtuins (Choudhary et al., 2014), is of great interest to pursue in the future.

Adenosine monophosphate-activated protein kinase (AMPK), another longevity mediator that is important for sensing low energy state, contribute to relieve the PER/CRY mediated circadian feedback repression. AMPK phosphorylates and activates casein kinase I epsilon (CKIε) for the subsequent phosphorylation of PER, therefore promote PER degradation (Um et al., 2007). In a similar action, AMPK phosphorylates CRY directly and facilitates CRY degradation (Lamia et al., 2009). The stimulation of AMPK activity leads to a phase advance effect. AMPK has been studied in many hypothalamic nuclei such as ARC, VMH and DMH, for energy balance and metabolism control (Lopez et al., 2016), yet the role in central circadian regulation in the SCN remains unclear.

The mammalian target of rapamycin (mTOR), an important sensor of insulin, growth factor, and mitogen inputs, has been revealed in circadian control through many effector proteins. For instance ribosomal S6 protein kinase 1 (S6K1), an important regulator of translation acting downstream of mTOR activation, can rhythmically phosphorylate BMAL1. The particular modification allows BMAL1 to work as a translation factor in a timely manner with response to the mTOR signaling, in addition to the canonical role in circadian transcription (Lipton et al., 2015). Of note, BMAL1 deficiency caused elevated activity of mTORC1 both in cell culture and *in vivo*. *In vivo* administration of the mTORC1 inhibitor Rapatar increased *Bmal1* null mice lifespan by 50% (Khapre et al., 2014), the results suggested complex, bi-directional regulations may exist between BMAL1 and mTOR. In the SCN, the photic signal activates mTOR signaling and promotes the translation of VIP by repressing 4E-BP1. Accordingly, the 4E-BP1 deficient mice exhibit accelerated re-entrainment upon light/dark shift and are more resilient to constant light mediated circadian disruption (Cao et al., 2013). Together, the findings reveal strong energy links among aging, metabolism and circadian physiology.

## CONCLUDING REMARKS

It is clear that age-related diseases such as cancer, type-2 diabetes, obesity and neurodegenerative disorders are profoundly metabolic-associated (Lopez-Otin et al., 2013), and functional circadian activities maybe key to preclude the abnormalities (Asher and Sassone-Corsi, 2015). Numerous data indicate nutrient-sensing/longevity pathways such as SIRT1 and others assist circadian control, and impose regulatory loops in coordinating photic and non-photic feeding stimuli. The beneficial effects offered viewpoints that interventions for promoting healthy aging and longevity may as well treat circadian disorders. Initiated trials on sirtuin-activating compounds (STACs) such as resveratrol, SRT2104 or NAD^+^ precursors (Bonkowski and Sinclair, 2016; Wood et al., 2004); metformin for AMPK activation (Barzilai et al., 2016); and rapamycin for mTOR inhibition (Harrison et al., 2009; Li et al., 2014) are suitable candidates for such interventions. Effects of these on central versus peripheral clocks, and the underlying mechanisms await careful analyses in the future.
